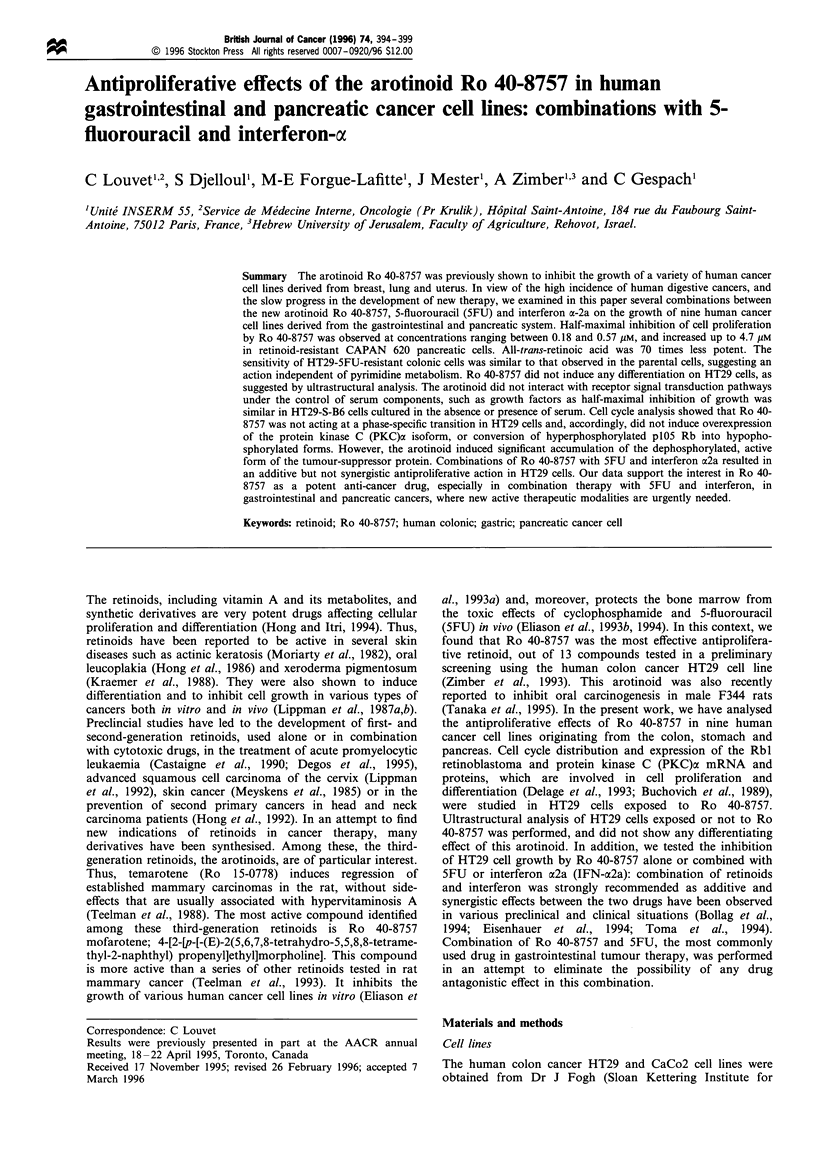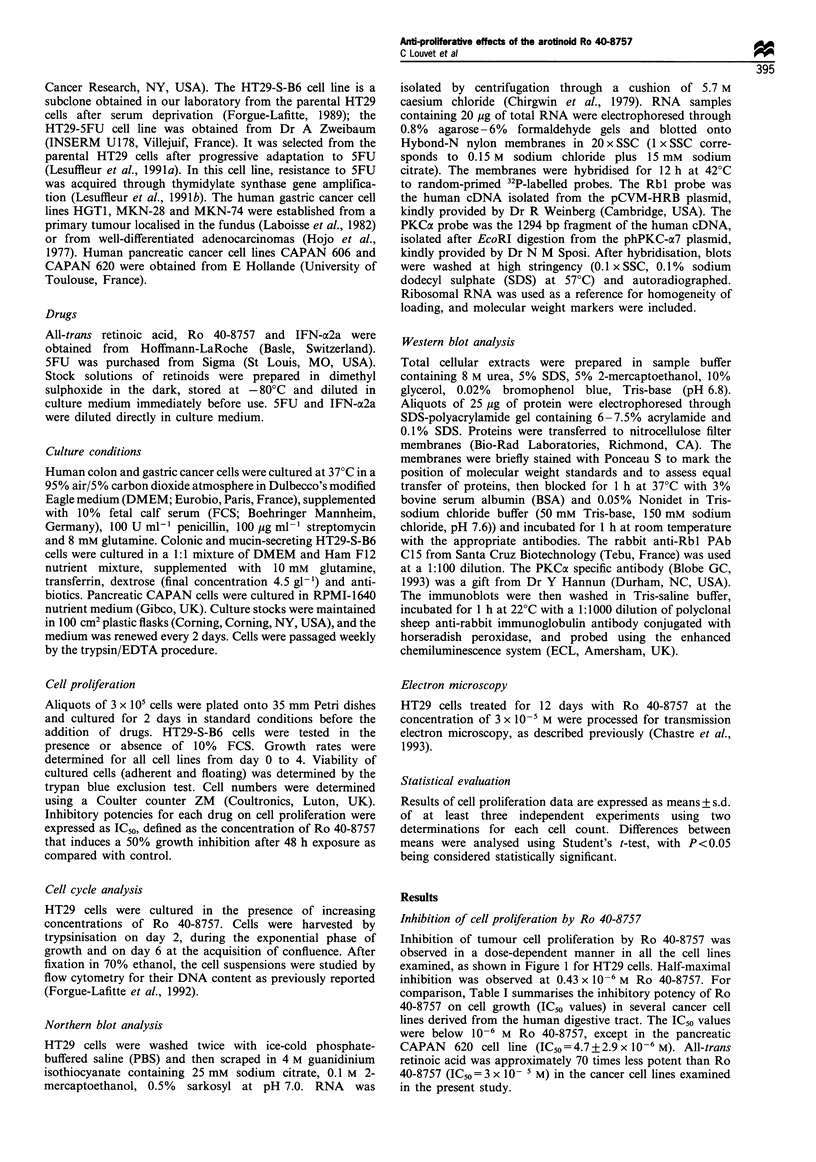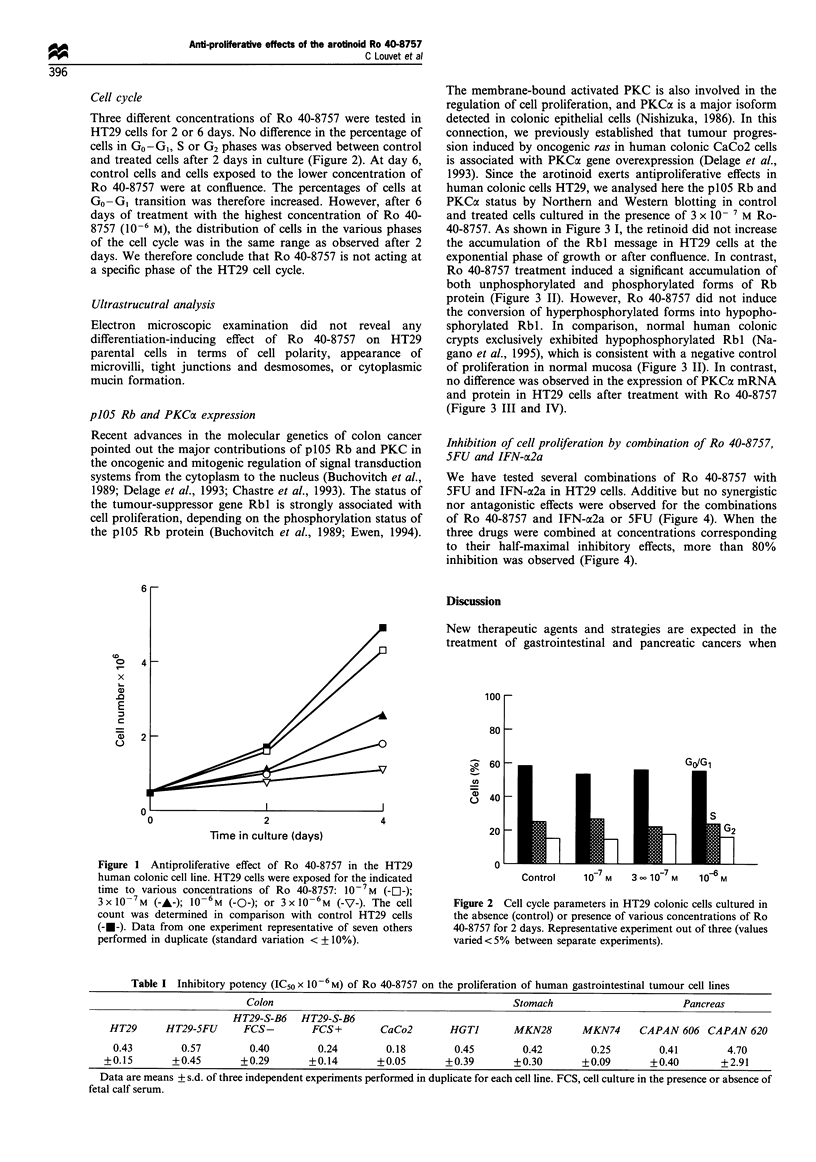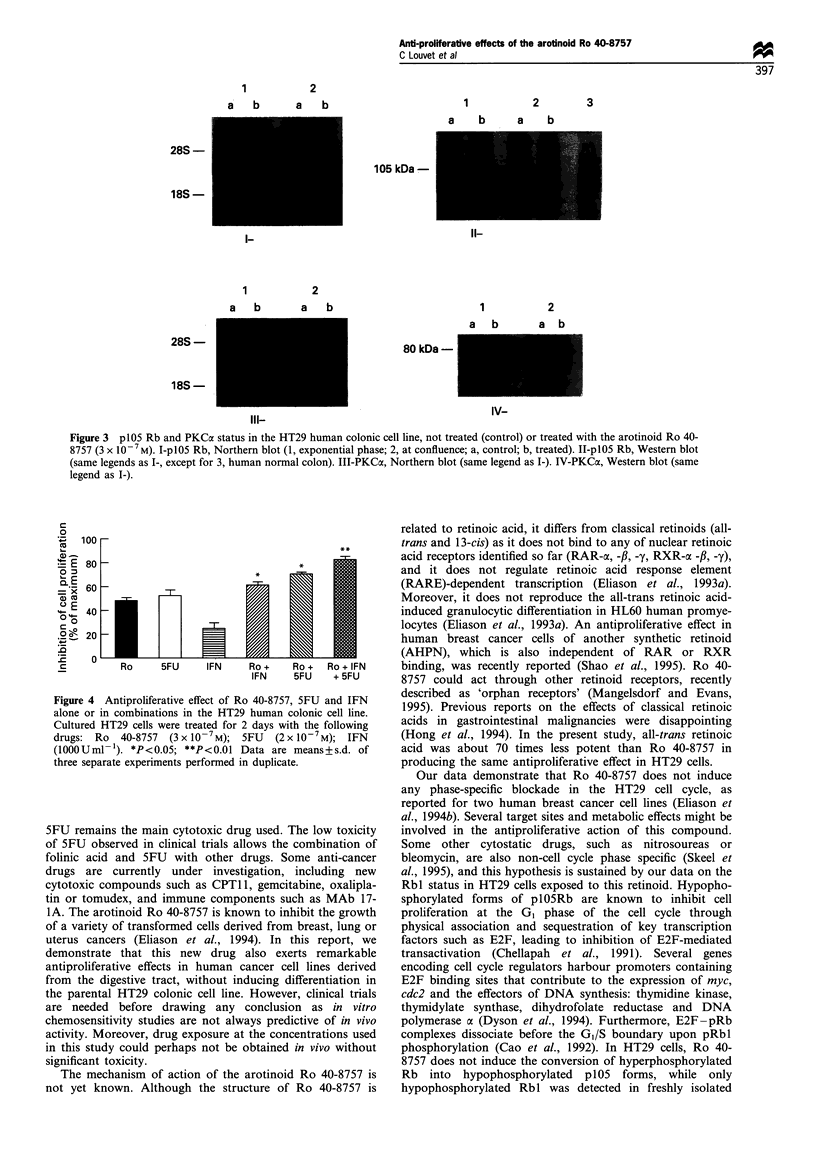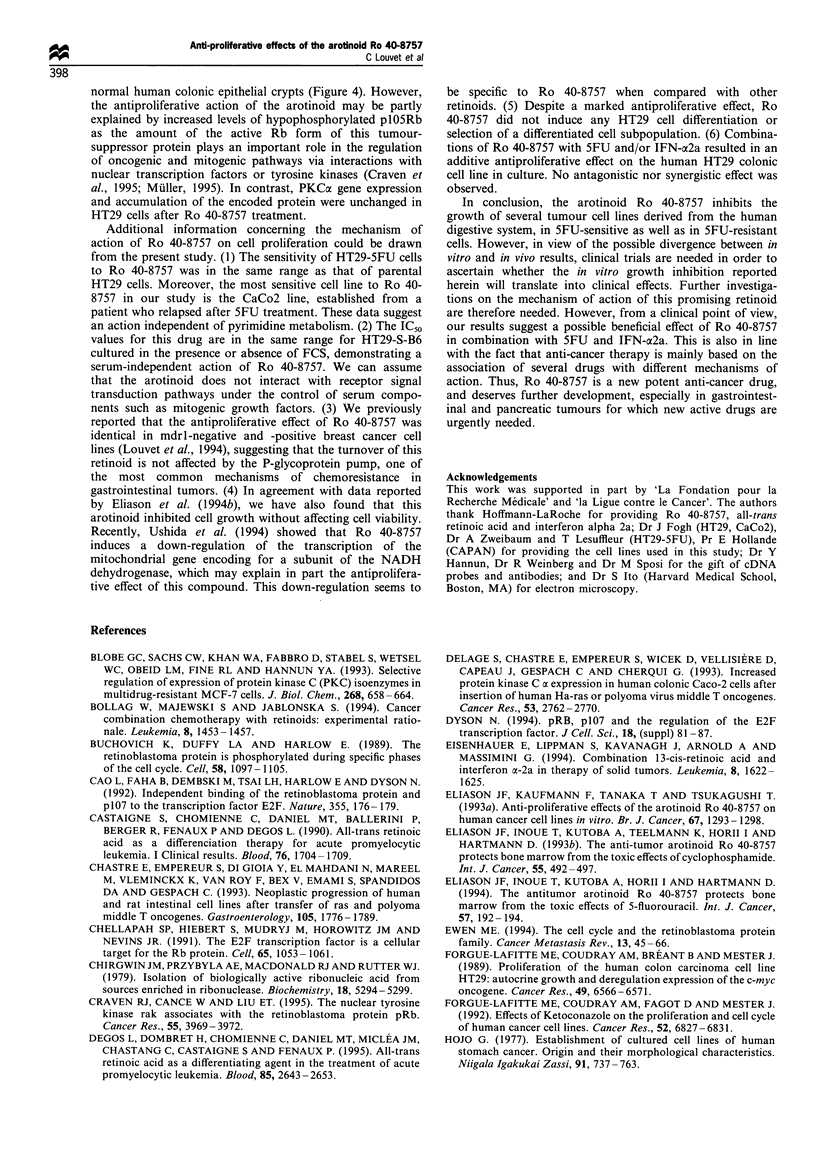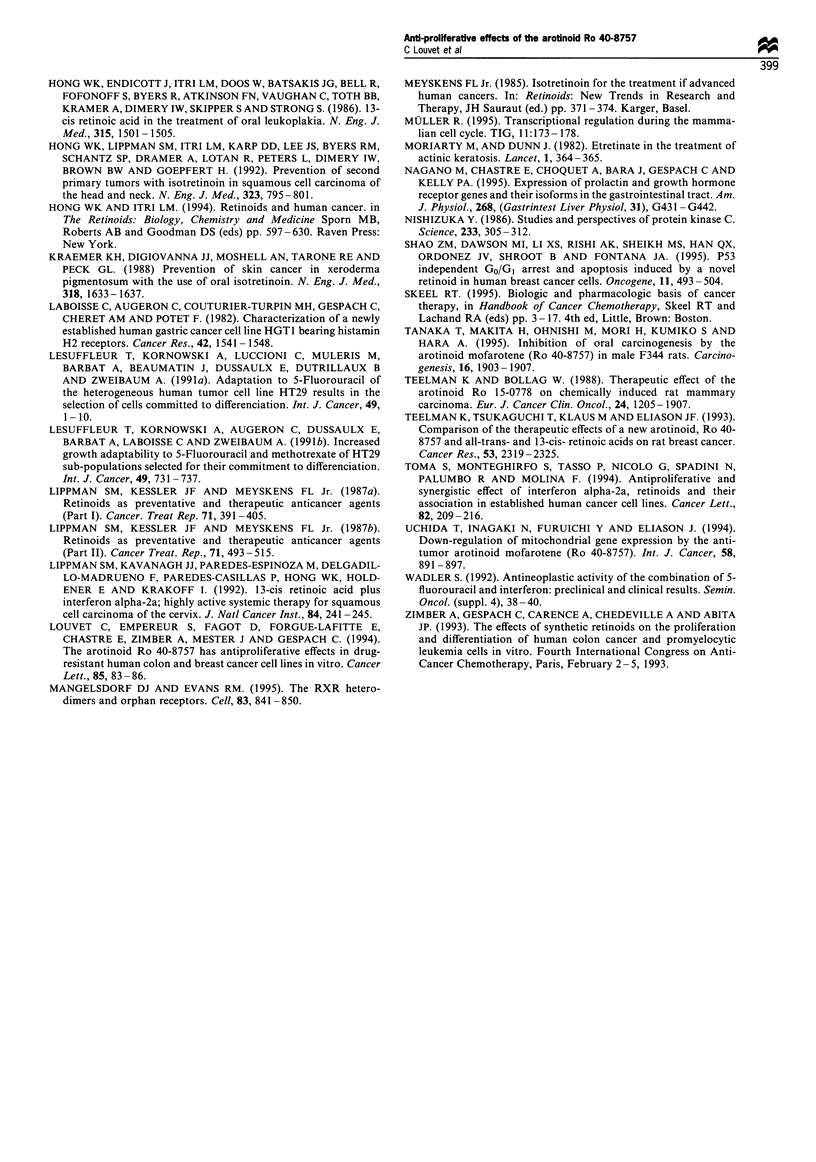# Antiproliferative effects of the arotinoid Ro 40-8757 in human gastrointestinal and pancreatic cancer cell lines: combinations with 5-fluorouracil and interferon-alpha.

**DOI:** 10.1038/bjc.1996.371

**Published:** 1996-08

**Authors:** C. Louvet, S. Djelloul, M. E. Forgue-Lafitte, J. Mester, A. Zimber, C. Gespach

**Affiliations:** Unité INSERM 55, Hôpital Saint-Antoine, Paris, France.

## Abstract

**Images:**


British Journal of Cancer (1996) 74, 394-399
© 1996 Stockton Press All rights reserved 0007-0920/96 $12.00

### Antiproliferative effects of the arotinoid Ro 40-8757 in human gastrointestinal and pancreatic cancer cell lines: combinations with 5-fluorouracil and interferon-α

C Louvet[1,2], S Djelloul[1], M-E Forgue-Lafitte[1], J Mester[1], A Zimber[1,3] and C Gespach[1]

[1]*Unité INSERM 55,* [2]*Service de Médecine Interne, Oncologie (Pr Krulik), Hôpital Saint-Antoine, 184 rue du Faubourg Saint-Antoine, 75012 Paris, France,* [3]*Hebrew University of Jerusalem, Faculty of Agriculture, Rehovot, Israel.*

**Summary** The arotinoid Ro 40-8757 was previously shown to inhibit the growth of a variety of human cancer cell lines derived from breast, lung and uterus. In view of the high incidence of human digestive cancers, and the slow progress in the development of new therapy, we examined in this paper several combinations between the new arotinoid Ro 40-8757, 5-fluorouracil (5FU) and interferon α-2a on the growth of nine human cancer cell lines derived from the gastrointestinal and pancreatic system. Half-maximal inhibition of cell proliferation by Ro 40-8757 was observed at concentrations ranging between 0.18 and 0.57 μM, and increased up to 4.7 μM in retinoid-resistant CAPAN 620 pancreatic cells. All-*trans*-retinoic acid was 70 times less potent. The sensitivity of HT29-5FU-resistant colonic cells was similar to that observed in the parental cells, suggesting an action independent of pyrimidine metabolism. Ro 40-8757 did not induce any differentiation on HT29 cells, as suggested by ultrastructural analysis. The arotinoid did not interact with receptor signal transduction pathways under the control of serum components, such as growth factors as half-maximal inhibition of growth was similar in HT29-S-B6 cells cultured in the absence or presence of serum. Cell cycle analysis showed that Ro 40-8757 was not acting at a phase-specific transition in HT29 cells and, accordingly, did not induce overexpression of the protein kinase C (PKC)α isoform, or conversion of hyperphosphorylated p105 Rb into hypophosphorylated forms. However, the arotinoid induced significant accumulation of the dephosphorylated, active form of the tumour-suppressor protein. Combinations of Ro 40-8757 with 5FU and interferon α2a resulted in an additive but not synergistic antiproliferative action in HT29 cells. Our data support the interest in Ro 40-8757 as a potent anti-cancer drug, especially in combination therapy with 5FU and interferon, in gastrointestinal and pancreatic cancers, where new active therapeutic modalities are urgently needed.

**Keywords:** retinoid; Ro 40-8757; human colonic; gastric; pancreatic cancer cell

The retinoids, including vitamin A and its metabolites, and synthetic derivatives are very potent drugs affecting cellular proliferation and differentiation (Hong and Itri, 1994). Thus, retinoids have been reported to be active in several skin diseases such as actinic keratosis (Moriarty *et al.*, 1982), oral leucoplakia (Hong *et al.*, 1986) and xeroderma pigmentosum (Kraemer *et al.*, 1988). They were also shown to induce differentiation and to inhibit cell growth in various types of cancers both *in vitro* and *in vivo* (Lippman *et al.*, 1987*a*,*b*). Preclincial studies have led to the development of first- and second-generation retinoids, used alone or in combination with cytotoxic drugs, in the treatment of acute promyelocytic leukaemia (Castaigne *et al.*, 1990; Degos *et al.*, 1995), advanced squamous cell carcinoma of the cervix (Lippman *et al.*, 1992), skin cancer (Meyskens *et al.*, 1985) or in the prevention of second primary cancers in head and neck carcinoma patients (Hong *et al.*, 1992). In an attempt to find new indications of retinoids in cancer therapy, many derivatives have been synthesised. Among these, the third-generation retinoids, the arotinoids, are of particular interest. Thus, temarotene (Ro 15-0778) induces regression of established mammary carcinomas in the rat, without side-effects that are usually associated with hypervitaminosis A (Teelman *et al.*, 1988). The most active compound identified among these third-generation retinoids is Ro 40-8757 mofarotene; 4-[2-[*p*-[-(E)-2(5,6,7,8-tetrahydro-5,5,8,8-tetramethyl-2-naphthyl) propenyl]ethyl]morpholine]. This compound is more active than a series of other retinoids tested in rat mammary cancer (Teelman *et al.*, 1993). It inhibits the growth of various human cancer cell lines *in vitro* (Eliason *et al.*, 1993*a*) and, moreover, protects the bone marrow from the toxic effects of cyclophosphamide and 5-fluorouracil (5FU) *in vivo* (Eliason *et al.*, 1993*b*, 1994). In this context, we found that Ro 40-8757 was the most effective antiproliferative retinoid, out of 13 compounds tested in a preliminary screening using the human colon cancer HT29 cell line (Zimber *et al.*, 1993). This arotinoid was also recently reported to inhibit oral carcinogenesis in male F344 rats (Tanaka *et al.*, 1995). In the present work, we have analysed the antiproliferative effects of Ro 40-8757 in nine human cancer cell lines originating from the colon, stomach and pancreas. Cell cycle distribution and expression of the Rb1 retinoblastoma and protein kinase C (PKC)α mRNA and proteins, which are involved in cell proliferation and differentiation (Delage *et al.*, 1993; Buchovich *et al.*, 1989), were studied in HT29 cells exposed to Ro 40-8757. Ultrastructural analysis of HT29 cells exposed or not to Ro 40-8757 was performed, and did not show any differentiating effect of this arotinoid. In addition, we tested the inhibition of HT29 cell growth by Ro 40-8757 alone or combined with 5FU or interferon α2a (IFN-α2a): combination of retinoids and interferon was strongly recommended as additive and synergistic effects between the two drugs have been observed in various preclinical and clinical situations (Bollag *et al.*, 1994; Eisenhauer *et al.*, 1994; Toma *et al.*, 1994). Combination of Ro 40-8757 and 5FU, the most commonly used drug in gastrointestinal tumour therapy, was performed in an attempt to eliminate the possibility of any drug antagonistic effect in this combination.

Correspondence: C Louvet
Results were previously presented in part at the AACR annual meeting, 18–22 April 1995, Toronto, Canada
Received 17 November 1995; revised 26 February 1996; accepted 7 March 1996

### Materials and methods

### *Cell lines*

The human colon cancer HT29 and CaCo2 cell lines were obtained from Dr J Fogh (Sloan Kettering Institute for

Cancer Research, NY, USA). The HT29-S-B6 cell line is a subclone obtained in our laboratory from the parental HT29 cells after serum deprivation (Forgue-Lafitte, 1989); the HT29-5FU cell line was obtained from Dr A Zweibaum (INSERM U178, Villejuif, France). It was selected from the parental HT29 cells after progressive adaptation to 5FU (Lesuffleur *et al*., 1991*a*). In this cell line, resistance to 5FU was acquired through thymidylate synthase gene amplification (Lesuffleur *et al*., 1991*b*). The human gastric cancer cell lines HGT1, MKN-28 and MKN-74 were established from a primary tumour localised in the fundus (Laboisse *et al*., 1982) or from well-differentiated adenocarcinomas (Hojo *et al*., 1977). Human pancreatic cancer cell lines CAPAN 606 and CAPAN 620 were obtained from E Hollande (University of Toulouse, France).

### *Drugs*

All-*trans* retinoic acid, Ro 40-8757 and IFN-α2a were obtained from Hoffmann-LaRoche (Basle, Switzerland). 5FU was purchased from Sigma (St Louis, MO, USA). Stock solutions of retinoids were prepared in dimethyl sulphoxide in the dark, stored at −80°C and diluted in culture medium immediately before use. 5FU and IFN-α2a were diluted directly in culture medium.

### *Culture conditions*

Human colon and gastric cancer cells were cultured at 37°C in a 95% air/5% carbon dioxide atmosphere in Dulbecco's modified Eagle medium (DMEM; Eurobio, Paris, France), supplemented with 10% fetal calf serum (FCS; Boehringer Mannheim, Germany), 100 U $ml^{-1}$ penicillin, 100 $\mu g$ $ml^{-1}$ streptomycin and 8 mM glutamine. Colonic and mucin-secreting HT29-S-B6 cells were cultured in a 1:1 mixture of DMEM and Ham F12 nutrient mixture, supplemented with 10 mM glutamine, transferrin, dextrose (final concentration 4.5 $gl^{-1}$) and antibiotics. Pancreatic CAPAN cells were cultured in RPMI-1640 nutrient medium (Gibco, UK). Culture stocks were maintained in 100 $cm^2$ plastic flasks (Corning, Corning, NY, USA), and the medium was renewed every 2 days. Cells were passaged weekly by the trypsin/EDTA procedure.

### *Cell proliferation*

Aliquots of $3 \times 10^5$ cells were plated onto 35 mm Petri dishes and cultured for 2 days in standard conditions before the addition of drugs. HT29-S-B6 cells were tested in the presence or absence of 10% FCS. Growth rates were determined for all cell lines from day 0 to 4. Viability of cultured cells (adherent and floating) was determined by the trypan blue exclusion test. Cell numbers were determined using a Coulter counter ZM (Coultronics, Luton, UK). Inhibitory potencies for each drug on cell proliferation were expressed as $IC_{50}$, defined as the concentration of Ro 40-8757 that induces a 50% growth inhibition after 48 h exposure as compared with control.

### *Cell cycle analysis*

HT29 cells were cultured in the presence of increasing concentrations of Ro 40-8757. Cells were harvested by trypsinisation on day 2, during the exponential phase of growth and on day 6 at the acquisition of confluence. After fixation in 70% ethanol, the cell suspensions were studied by flow cytometry for their DNA content as previously reported (Forgue-Lafitte *et al*., 1992).

### *Northern blot analysis*

HT29 cells were washed twice with ice-cold phosphate-buffered saline (PBS) and then scraped in 4 M guanidinium isothiocyanate containing 25 mM sodium citrate, 0.1 M 2-mercaptoethanol, 0.5% sarkosyl at pH 7.0. RNA was isolated by centrifugation through a cushion of 5.7 M caesium chloride (Chirgwin *et al*., 1979). RNA samples containing 20 $\mu g$ of total RNA were electrophoresed through 0.8% agarose–6% formaldehyde gels and blotted onto Hybond-N nylon membranes in 20 × SSC (1 × SSC corresponds to 0.15 M sodium chloride plus 15 mM sodium citrate). The membranes were hybridised for 12 h at 42°C to random-primed $^{32}P$-labelled probes. The Rb1 probe was the human cDNA isolated from the pCVM-HRB plasmid, kindly provided by Dr R Weinberg (Cambridge, USA). The PKCα probe was the 1294 bp fragment of the human cDNA, isolated after *Eco*RI digestion from the phPKC-α7 plasmid, kindly provided by Dr N M Sposi. After hybridisation, blots were washed at high stringency (0.1 × SSC, 0.1% sodium dodecyl sulphate (SDS) at 57°C) and autoradiographed. Ribosomal RNA was used as a reference for homogeneity of loading, and molecular weight markers were included.

### *Western blot analysis*

Total cellular extracts were prepared in sample buffer containing 8 M urea, 5% SDS, 5% 2-mercaptoethanol, 10% glycerol, 0.02% bromophenol blue, Tris-base (pH 6.8). Aliquots of 25 $\mu g$ of protein were electrophoresed through SDS-polyacrylamide gel containing 6–7.5% acrylamide and 0.1% SDS. Proteins were transferred to nitrocellulose filter membranes (Bio-Rad Laboratories, Richmond, CA). The membranes were briefly stained with Ponceau S to mark the position of molecular weight standards and to assess equal transfer of proteins, then blocked for 1 h at 37°C with 3% bovine serum albumin (BSA) and 0.05% Nonidet in Tris-sodium chloride buffer (50 mM Tris-base, 150 mM sodium chloride, pH 7.6)) and incubated for 1 h at room temperature with the appropriate antibodies. The rabbit anti-Rb1 PAb C15 from Santa Cruz Biotechnology (Tebu, France) was used at a 1:100 dilution. The PKCα specific antibody (Blobe GC, 1993) was a gift from Dr Y Hannun (Durham, NC, USA). The immunoblots were then washed in Tris-saline buffer, incubated for 1 h at 22°C with a 1:1000 dilution of polyclonal sheep anti-rabbit immunoglobulin antibody conjugated with horseradish peroxidase, and probed using the enhanced chemiluminescence system (ECL, Amersham, UK).

### *Electron microscopy*

HT29 cells treated for 12 days with Ro 40-8757 at the concentration of $3 \times 10^{-5}$ M were processed for transmission electron microscopy, as described previously (Chastre *et al*., 1993).

### *Statistical evaluation*

Results of cell proliferation data are expressed as means ± s.d. of at least three independent experiments using two determinations for each cell count. Differences between means were analysed using Student's *t*-test, with $P < 0.05$ being considered statistically significant.

### Results

### *Inhibition of cell proliferation by Ro 40-8757*

Inhibition of tumour cell proliferation by Ro 40-8757 was observed in a dose-dependent manner in all the cell lines examined, as shown in Figure 1 for HT29 cells. Half-maximal inhibition was observed at $0.43 \times 10^{-6}$ M Ro 40-8757. For comparison, Table I summarises the inhibitory potency of Ro 40-8757 on cell growth ($IC_{50}$ values) in several cancer cell lines derived from the human digestive tract. The $IC_{50}$ values were below $10^{-6}$ M Ro 40-8757, except in the pancreatic CAPAN 620 cell line ($IC_{50} = 4.7 \pm 2.9 \times 10^{-6}$ M). All-*trans* retinoic acid was approximately 70 times less potent than Ro 40-8757 ($IC_{50} = 3 \times 10^{-5}$ M) in the cancer cell lines examined in the present study.

*Cell cycle*

Three different concentrations of Ro 40-8757 were tested in HT29 cells for 2 or 6 days. No difference in the percentage of cells in $G_0-G_1$, S or $G_2$ phases was observed between control and treated cells after 2 days in culture (Figure 2). At day 6, control cells and cells exposed to the lower concentration of Ro 40-8757 were at confluence. The percentages of cells at $G_0-G_1$ transition was therefore increased. However, after 6 days of treatment with the highest concentration of Ro 40-8757 ($10^{-6}$ M), the distribution of cells in the various phases of the cell cycle was in the same range as observed after 2 days. We therefore conclude that Ro 40-8757 is not acting at a specific phase of the HT29 cell cycle.

*Ultrastrucutral analysis*

Electron microscopic examination did not reveal any differentiation-inducing effect of Ro 40-8757 on HT29 parental cells in terms of cell polarity, appearance of microvilli, tight junctions and desmosomes, or cytoplasmic mucin formation.

*p105 Rb and PKCα expression*

Recent advances in the molecular genetics of colon cancer pointed out the major contributions of p105 Rb and PKC in the oncogenic and mitogenic regulation of signal transduction systems from the cytoplasm to the nucleus (Buchovitch *et al.*, 1989; Delage *et al.*, 1993; Chastre *et al.*, 1993). The status of the tumour-suppressor gene Rb1 is strongly associated with cell proliferation, depending on the phosphorylation status of the p105 Rb protein (Buchovitch *et al.*, 1989; Ewen, 1994).

**Figure 1** Antiproliferative effect of Ro 40-8757 in the HT29 human colonic cell line. HT29 cells were exposed for the indicated time to various concentrations of Ro 40-8757: $10^{-7}$M (-□-); $3 \times 10^{-7}$M (-▲-); $10^{-6}$M (-○-); or $3 \times 10^{-6}$M (-▽-). The cell count was determined in comparison with control HT29 cells (-■-). Data from one experiment representative of seven others performed in duplicate (standard variation $< \pm 10\%$).

The membrane-bound activated PKC is also involved in the regulation of cell proliferation, and PKCα is a major isoform detected in colonic epithelial cells (Nishizuka, 1986). In this connection, we previously established that tumour progression induced by oncogenic *ras* in human colonic CaCo2 cells is associated with PKCα gene overexpression (Delage *et al.*, 1993). Since the arotinoid exerts antiproliferative effects in human colonic cells HT29, we analysed here the p105 Rb and PKCα status by Northern and Western blotting in control and treated cells cultured in the presence of $3 \times 10^{-7}$ M Ro-40-8757. As shown in Figure 3 I, the retinoid did not increase the accumulation of the Rb1 message in HT29 cells at the exponential phase of growth or after confluence. In contrast, Ro 40-8757 treatment induced a significant accumulation of both unphosphorylated and phosphorylated forms of Rb protein (Figure 3 II). However, Ro 40-8757 did not induce the conversion of hyperphosphorylated forms into hypophosphorylated Rb1. In comparison, normal human colonic crypts exclusively exhibited hypophosphorylated Rb1 (Nagano *et al.*, 1995), which is consistent with a negative control of proliferation in normal mucosa (Figure 3 II). In contrast, no difference was observed in the expression of PKCα mRNA and protein in HT29 cells after treatment with Ro 40-8757 (Figure 3 III and IV).

*Inhibition of cell proliferation by combination of Ro 40-8757, 5FU and IFN-α2a*

We have tested several combinations of Ro 40-8757 with 5FU and IFN-α2a in HT29 cells. Additive but no synergistic nor antagonistic effects were observed for the combinations of Ro 40-8757 and IFN-α2a or 5FU (Figure 4). When the three drugs were combined at concentrations corresponding to their half-maximal inhibitory effects, more than 80% inhibition was observed (Figure 4).

### Discussion

New therapeutic agents and strategies are expected in the treatment of gastrointestinal and pancreatic cancers when

**Figure 2** Cell cycle parameters in HT29 colonic cells cultured in the absence (control) or presence of various concentrations of Ro 40-8757 for 2 days. Representative experiment out of three (values varied < 5% between separate experiments).

**Table I** Inhibitory potency ($IC_{50} \times 10^{-6}$M) of Ro 40-8757 on the proliferation of human gastrointestinal tumour cell lines

| *Colon* | | | | | *Stomach* | | | *Pancreas* | |
|---|---|---|---|---|---|---|---|---|---|
| *HT29* | *HT29-5FU* | *HT29-S-B6 FCS−* | *HT29-S-B6 FCS+* | *CaCo2* | *HGT1* | *MKN28* | *MKN74* | *CAPAN 606* | *CAPAN 620* |
| 0.43 ±0.15 | 0.57 ±0.45 | 0.40 ±0.29 | 0.24 ±0.14 | 0.18 ±0.05 | 0.45 ±0.39 | 0.42 ±0.30 | 0.25 ±0.09 | 0.41 ±0.40 | 4.70 ±2.91 |

Data are means ± s.d. of three independent experiments performed in duplicate for each cell line. FCS, cell culture in the presence or absence of fetal calf serum.

**Figure 3** p105 Rb and PKCα status in the HT29 human colonic cell line, not treated (control) or treated with the arotinoid Ro 40-8757 ($3 \times 10^{-7}$ M). I-p105 Rb, Northern blot (1, exponential phase; 2, at confluence; a, control; b, treated). II-p105 Rb, Western blot (same legends as I-, except for 3, human normal colon). III-PKCα, Northern blot (same legend as I-). IV-PKCα, Western blot (same legend as I-).

**Figure 4** Antiproliferative effect of Ro 40-8757, 5FU and IFN alone or in combinations in the HT29 human colonic cell line. Cultured HT29 cells were treated for 2 days with the following drugs: Ro 40-8757 ($3 \times 10^{-7}$ M); 5FU ($2 \times 10^{-7}$ M); IFN (1000 U ml$^{-1}$). *$P<0.05$; **$P<0.01$ Data are means±s.d. of three separate experiments performed in duplicate.

5FU remains the main cytotoxic drug used. The low toxicity of 5FU observed in clinical trials allows the combination of folinic acid and 5FU with other drugs. Some anti-cancer drugs are currently under investigation, including new cytotoxic compounds such as CPT11, gemcitabine, oxaliplatin or tomudex, and immune components such as MAb 17-1A. The arotinoid Ro 40-8757 is known to inhibit the growth of a variety of transformed cells derived from breast, lung or uterus cancers (Eliason *et al.*, 1994). In this report, we demonstrate that this new drug also exerts remarkable antiproliferative effects in human cancer cell lines derived from the digestive tract, without inducing differentiation in the parental HT29 colonic cell line. However, clinical trials are needed before drawing any conclusion as *in vitro* chemosensitivity studies are not always predictive of *in vivo* activity. Moreover, drug exposure at the concentrations used in this study could perhaps not be obtained *in vivo* without significant toxicity.

The mechanism of action of the arotinoid Ro 40-8757 is not yet known. Although the structure of Ro 40-8757 is related to retinoic acid, it differs from classical retinoids (all-*trans* and 13-*cis*) as it does not bind to any of nuclear retinoic acid receptors identified so far (RAR-α, -β, -γ, RXR-α -β, -γ), and it does not regulate retinoic acid response element (RARE)-dependent transcription (Eliason *et al.*, 1993*a*). Moreover, it does not reproduce the all-trans retinoic acid-induced granulocytic differentiation in HL60 human promyelocytes (Eliason *et al.*, 1993*a*). An antiproliferative effect in human breast cancer cells of another synthetic retinoid (AHPN), which is also independent of RAR or RXR binding, was recently reported (Shao *et al.*, 1995). Ro 40-8757 could act through other retinoid receptors, recently described as 'orphan receptors' (Mangelsdorf and Evans, 1995). Previous reports on the effects of classical retinoic acids in gastrointestinal malignancies were disappointing (Hong *et al.*, 1994). In the present study, all-*trans* retinoic acid was about 70 times less potent than Ro 40-8757 in producing the same antiproliferative effect in HT29 cells.

Our data demonstrate that Ro 40-8757 does not induce any phase-specific blockade in the HT29 cell cycle, as reported for two human breast cancer cell lines (Eliason *et al.*, 1994*b*). Several target sites and metabolic effects might be involved in the antiproliferative action of this compound. Some other cytostatic drugs, such as nitrosoureas or bleomycin, are also non-cell cycle phase specific (Skeel *et al.*, 1995), and this hypothesis is sustained by our data on the Rb1 status in HT29 cells exposed to this retinoid. Hypophosphorylated forms of p105Rb are known to inhibit cell proliferation at the $G_1$ phase of the cell cycle through physical association and sequestration of key transcription factors such as E2F, leading to inhibition of E2F-mediated transactivation (Chellapah *et al.*, 1991). Several genes encoding cell cycle regulators harbour promoters containing E2F binding sites that contribute to the expression of *myc*, *cdc2* and the effectors of DNA synthesis: thymidine kinase, thymidylate synthase, dihydrofolate reductase and DNA polymerase α (Dyson *et al.*, 1994). Furthermore, E2F–pRb complexes dissociate before the $G_1$/S boundary upon pRb1 phosphorylation (Cao *et al.*, 1992). In HT29 cells, Ro 40-8757 does not induce the conversion of hyperphosphorylated Rb into hypophosphorylated p105 forms, while only hypophosphorylated Rb1 was detected in freshly isolated normal human colonic epithelial crypts (Figure 4). However, the antiproliferative action of the arotinoid may be partly explained by increased levels of hypophosphorylated p105Rb as the amount of the active Rb form of this tumour-suppressor protein plays an important role in the regulation of oncogenic and mitogenic pathways via interactions with nuclear transcription factors or tyrosine kinases (Craven *et al.*, 1995; Müller, 1995). In contrast, PKC$\alpha$ gene expression and accumulation of the encoded protein were unchanged in HT29 cells after Ro 40-8757 treatment.

Additional information concerning the mechanism of action of Ro 40-8757 on cell proliferation could be drawn from the present study. (1) The sensitivity of HT29-5FU cells to Ro 40-8757 was in the same range as that of parental HT29 cells. Moreover, the most sensitive cell line to Ro 40-8757 in our study is the CaCo2 line, established from a patient who relapsed after 5FU treatment. These data suggest an action independent of pyrimidine metabolism. (2) The $IC_{50}$ values for this drug are in the same range for HT29-S-B6 cultured in the presence or absence of FCS, demonstrating a serum-independent action of Ro 40-8757. We can assume that the arotinoid does not interact with receptor signal transduction pathways under the control of serum components such as mitogenic growth factors. (3) We previously reported that the antiproliferative effect of Ro 40-8757 was identical in mdr1-negative and -positive breast cancer cell lines (Louvet *et al.*, 1994), suggesting that the turnover of this retinoid is not affected by the P-glycoprotein pump, one of the most common mechanisms of chemoresistance in gastrointestinal tumors. (4) In agreement with data reported by Eliason *et al.* (1994*b*), we have also found that this arotinoid inhibited cell growth without affecting cell viability. Recently, Ushida *et al.* (1994) showed that Ro 40-8757 induces a down-regulation of the transcription of the mitochondrial gene encoding for a subunit of the NADH dehydrogenase, which may explain in part the antiproliferative effect of this compound. This down-regulation seems to be specific to Ro 40-8757 when compared with other retinoids. (5) Despite a marked antiproliferative effect, Ro 40-8757 did not induce any HT29 cell differentiation or selection of a differentiated cell subpopulation. (6) Combinations of Ro 40-8757 with 5FU and/or IFN-$\alpha$2a resulted in an additive antiproliferative effect on the human HT29 colonic cell line in culture. No antagonistic nor synergistic effect was observed.

In conclusion, the arotinoid Ro 40-8757 inhibits the growth of several tumour cell lines derived from the human digestive system, in 5FU-sensitive as well as in 5FU-resistant cells. However, in view of the possible divergence between *in vitro* and *in vivo* results, clinical trials are needed in order to ascertain whether the *in vitro* growth inhibition reported herein will translate into clinical effects. Further investigations on the mechanism of action of this promising retinoid are therefore needed. However, from a clinical point of view, our results suggest a possible beneficial effect of Ro 40-8757 in combination with 5FU and IFN-$\alpha$2a. This is also in line with the fact that anti-cancer therapy is mainly based on the association of several drugs with different mechanisms of action. Thus, Ro 40-8757 is a new potent anti-cancer drug, and deserves further development, especially in gastrointestinal and pancreatic tumours for which new active drugs are urgently needed.

**Acknowledgements**

This work was supported in part by 'La Fondation pour la Recherche Médicale' and 'la Ligue contre le Cancer'. The authors thank Hoffmann-LaRoche for providing Ro 40-8757, all-*trans* retinoic acid and interferon alpha 2a; Dr J Fogh (HT29, CaCo2), Dr A Zweibaum and T Lesuffleur (HT29-5FU), Pr E Hollande (CAPAN) for providing the cell lines used in this study; Dr Y Hannun, Dr R Weinberg and Dr M Sposi for the gift of cDNA probes and antibodies; and Dr S Ito (Harvard Medical School, Boston, MA) for electron microscopy.

### References

BLOBE GC, SACHS CW, KHAN WA, FABBRO D, STABEL S, WETSEL WC, OBEID LM, FINE RL AND HANNUN YA. (1993). Selective regulation of expression of protein kinase C (PKC) isoenzymes in multidrug-resistant MCF-7 cells. *J. Biol. Chem.*, **268**, 658–664.

BOLLAG W, MAJEWSKI S AND JABLONSKA S. (1994). Cancer combination chemotherapy with retinoids: experimental rationale. *Leukemia*, **8**, 1453–1457.

BUCHOVICH K, DUFFY LA AND HARLOW E. (1989). The retinoblastoma protein is phosphorylated during specific phases of the cell cycle. *Cell*, **58**, 1097–1105.

CAO L, FAHA B, DEMBSKI M, TSAI LH, HARLOW E AND DYSON N. (1992). Independent binding of the retinoblastoma protein and p107 to the transcription factor E2F. *Nature*, 355, 176–179.

CASTAIGNE S, CHOMIENNE C, DANIEL MT, BALLERINI P, BERGER R, FENAUX P AND DEGOS L. (1990). All-trans retinoic acid as a differenciation therapy for acute promyelocytic leukemia. I Clinical results. *Blood*, **76**, 1704–1709.

CHASTRE E, EMPEREUR S, DI GIOIA Y, EL MAHDANI N, MAREEL M, VLEMINCKX K, VAN ROY F, BEX V, EMAMI S, SPANDIDOS DA AND GESPACH C. (1993). Neoplastic progression of human and rat intestinal cell lines after transfer of ras and polyoma middle T oncogenes. *Gastroenterology*, **105**, 1776–1789.

CHELLAPAH SP, HIEBERT S, MUDRYJ M, HOROWITZ JM AND NEVINS JR. (1991). The E2F transcription factor is a cellular target for the Rb protein. *Cell*, **65**, 1053–1061.

CHIRGWIN JM, PRZYBYLA AE, MACDONALD RJ AND RUTTER WJ. (1979). Isolation of biologically active ribonucleic acid from sources enriched in ribonuclease. *Biochemistry*, **18**, 5294–5299.

CRAVEN RJ, CANCE W AND LIU ET. (1995). The nuclear tyrosine kinase rak associates with the retinoblastoma protein pRb. *Cancer Res.*, **55**, 3969–3972.

DEGOS L, DOMBRET H, CHOMIENNE C, DANIEL MT, MICLÉA JM, CHASTANG C, CASTAIGNE S AND FENAUX P. (1995). All-trans retinoic acid as a differentiating agent in the treatment of acute promyelocytic leukemia. *Blood*, **85**, 2643–2653.

DELAGE S, CHASTRE E, EMPEREUR S, WICEK D, VELLISIÈRE D, CAPEAU J, GESPACH C AND CHERQUI G. (1993). Increased protein kinase C $\alpha$ expression in human colonic Caco-2 cells after insertion of human Ha-ras or polyoma virus middle T oncogenes. *Cancer Res.*, **53**, 2762–2770.

DYSON N. (1994). pRB, p107 and the regulation of the E2F transcription factor. *J Cell. Sci.*, **18**, (suppl) 81–87.

EISENHAUER E, LIPPMAN S, KAVANAGH J, ARNOLD A AND MASSIMINI G. (1994). Combination 13-cis-retinoic acid and interferon $\alpha$-2a in therapy of solid tumors. *Leukemia*, **8**, 1622–1625.

ELIASON JF, KAUFMANN F, TANAKA T AND TSUKAGUSHI T. (1993*a*). Anti-proliferative effects of the arotinoid Ro 40-8757 on human cancer cell lines *in vitro*. *Br. J. Cancer*, **67**, 1293–1298.

ELIASON JF, INOUE T, KUTOBA A, TEELMANN K, HORII I AND HARTMANN D. (1993*b*). The anti-tumor arotinoid Ro 40-8757 protects bone marrow from the toxic effects of cyclophosphamide. *Int. J. Cancer*, **55**, 492–497.

ELIASON JF, INOUE T, KUTOBA A, HORII I AND HARTMANN D. (1994). The antitumor arotinoid Ro 40-8757 protects bone marrow from the toxic effects of 5-fluorouracil. *Int. J. Cancer*, **57**, 192–194.

EWEN ME. (1994). The cell cycle and the retinoblastoma protein family. *Cancer Metastasis Rev.*, **13**, 45–66.

FORGUE-LAFITTE ME, COUDRAY AM, BRÉANT B AND MESTER J. (1989). Proliferation of the human colon carcinoma cell line HT29: autocrine growth and deregulation expression of the c-*myc* oncogene. *Cancer Res.*, **49**, 6566–6571.

FORGUE-LAFITTE ME, COUDRAY AM, FAGOT D AND MESTER J. (1992). Effects of Ketoconazole on the proliferation and cell cycle of human cancer cell lines. *Cancer Res.*, **52**, 6827–6831.

HOJO G. (1977). Establishment of cultured cell lines of human stomach cancer. Origin and their morphological characteristics. *Niigala Igakukai Zassi*, **91**, 737–763.

HONG WK, ENDICOTT J, ITRI LM, DOOS W, BATSAKIS JG, BELL R, FOFONOFF S, BYERS R, ATKINSON FN, VAUGHAN C, TOTH BB, KRAMER A, DIMERY IW, SKIPPER S AND STRONG S. (1986). 13-cis retinoic acid in the treatment of oral leukoplakia. *N. Eng. J. Med.*, **315**, 1501–1505.

HONG WK, LIPPMAN SM, ITRI LM, KARP DD, LEE JS, BYERS RM, SCHANTZ SP, DRAMER A, LOTAN R, PETERS L, DIMERY IW, BROWN BW AND GOEPFERT H. (1992). Prevention of second primary tumors with isotretinoin in squamous cell carcinoma of the head and neck. *N. Eng. J. Med.*, **323**, 795–801.

HONG WK AND ITRI LM. (1994). Retinoids and human cancer. in *The Retinoids: Biology, Chemistry and Medicine* Sporn MB, Roberts AB and Goodman DS (eds) pp. 597–630. Raven Press: New York.

KRAEMER KH, DIGIOVANNA JJ, MOSHELL AN, TARONE RE AND PECK GL. (1988) Prevention of skin cancer in xeroderma pigmentosum with the use of oral isotretinoin. *N. Eng. J. Med.*, **318**, 1633–1637.

LABOISSE C, AUGERON C, COUTURIER-TURPIN MH, GESPACH C, CHERET AM AND POTET F. (1982). Characterization of a newly established human gastric cancer cell line HGT1 bearing histamin H2 receptors. *Cancer Res.*, **42**, 1541–1548.

LESUFFLEUR T, KORNOWSKI A, LUCCIONI C, MULERIS M, BARBAT A, BEAUMATIN J, DUSSAULX E, DUTRILLAUX B AND ZWEIBAUM A. (1991*a*). Adaptation to 5-Fluorouracil of the heterogeneous human tumor cell line HT29 results in the selection of cells committed to differenciation. *Int. J. Cancer*, **49**, 1–10.

LESUFFLEUR T, KORNOWSKI A, AUGERON C, DUSSAULX E, BARBAT A, LABOISSE C AND ZWEIBAUM A. (1991*b*). Increased growth adaptability to 5-Fluorouracil and methotrexate of HT29 sub-populations selected for their commitment to differenciation. *Int. J. Cancer*, **49**, 731–737.

LIPPMAN SM, KESSLER JF AND MEYSKENS FL Jr. (1987*a*). Retinoids as preventative and therapeutic anticancer agents (Part I). *Cancer. Treat Rep.* **71**, 391–405.

LIPPMAN SM, KESSLER JF AND MEYSKENS FL Jr. (1987*b*). Retinoids as preventative and therapeutic anticancer agents (Part II). *Cancer Treat. Rep.*, **71**, 493–515.

LIPPMAN SM, KAVANAGH JJ, PAREDES-ESPINOZA M, DELGADILLO-MADRUENO F, PAREDES-CASILLAS P, HONG WK, HOLDENER E AND KRAKOFF I. (1992). 13-cis retinoic acid plus interferon alpha-2a; highly active systemic therapy for squamous cell carcinoma of the cervix. *J. Natl Cancer Inst.*, **84**, 241–245.

LOUVET C, EMPEREUR S, FAGOT D, FORGUE-LAFITTE E, CHASTRE E, ZIMBER A, MESTER J AND GESPACH C. (1994). The arotinoid Ro 40-8757 has antiproliferative effects in drug-resistant human colon and breast cancer cell lines in vitro. *Cancer Lett.*, **85**, 83–86.

MANGELSDORF DJ AND EVANS RM. (1995). The RXR heterodimers and orphan receptors. *Cell*, **83**, 841–850.

MEYSKENS FL Jr. (1985). Isotretinoin for the treatment if advanced human cancers. In: *Retinoids*: New Trends in Research and Therapy, JH Sauraut (ed.) pp. 371–374. Karger, Basel.

MÜLLER R. (1995). Transcriptional regulation during the mammalian cell cycle. TIG, 11:173–178.

MORIARTY M, AND DUNN J. (1982). Etretinate in the treatment of actinic keratosis. *Lancet*, **1**, 364–365.

NAGANO M, CHASTRE E, CHOQUET A, BARA J, GESPACH C AND KELLY PA. (1995). Expression of prolactin and growth hormone receptor genes and their isoforms in the gastrointestinal tract. *Am. J. Physiol.*, **268**, (*Gastrintest Liver Physiol*, **31**), G431–G442.

NISHIZUKA Y. (1986). Studies and perspectives of protein kinase C. *Science*, **233**, 305–312.

SHAO ZM, DAWSON MI, LI XS, RISHI AK, SHEIKH MS, HAN QX, ORDONEZ JV, SHROOT B AND FONTANA JA. (1995). P53 independent $G_0/G_1$ arrest and apoptosis induced by a novel retinoid in human breast cancer cells. *Oncogene*, **11**, 493–504.

SKEEL RT. (1995). Biologic and pharmacologic basis of cancer therapy, in *Handbook of Cancer Chemotherapy*, Skeel RT and Lachand RA (eds) pp. 3–17. 4th ed, Little, Brown: Boston.

TANAKA T, MAKITA H, OHNISHI M, MORI H, KUMIKO S AND HARA A. (1995). Inhibition of oral carcinogenesis by the arotinoid mofarotene (Ro 40-8757) in male F344 rats. *Carcinogenesis*, **16**, 1903–1907.

TEELMAN K AND BOLLAG W. (1988). Therapeutic effect of the arotinoid Ro 15-0778 on chemically induced rat mammary carcinoma. *Eur. J. Cancer Clin. Oncol.*, **24**, 1205–1907.

TEELMAN K, TSUKAGUCHI T, KLAUS M AND ELIASON JF. (1993). Comparison of the therapeutic effects of a new arotinoid, Ro 40-8757 and all-trans- and 13-cis- retinoic acids on rat breast cancer. *Cancer Res.*, **53**, 2319–2325.

TOMA S, MONTEGHIRFO S, TASSO P, NICOLO G, SPADINI N, PALUMBO R AND MOLINA F. (1994). Antiproliferative and synergistic effect of interferon alpha-2a, retinoids and their association in established human cancer cell lines. *Cancer Lett.*, **82**, 209–216.

UCHIDA T, INAGAKI N, FURUICHI Y AND ELIASON J. (1994). Down-regulation of mitochondrial gene expression by the antitumor arotinoid mofarotene (Ro 40-8757). *Int. J. Cancer*, **58**, 891–897.

WADLER S. (1992). Antineoplastic activity of the combination of 5-fluorouracil and interferon: preclinical and clinical results. *Semin. Oncol.* (suppl. 4), 38–40.

ZIMBER A, GESPACH C, CARENCE A, CHEDEVILLE A AND ABITA JP. (1993). The effects of synthetic retinoids on the proliferation and differentiation of human colon cancer and promyelocytic leukemia cells in vitro. Fourth International Congress on Anti-Cancer Chemotherapy, Paris, February 2–5, 1993.